# MAFB-mediated CEBPA regulated human urothelium growth through Wnt/β-catenin signaling pathway

**DOI:** 10.1016/j.gendis.2024.101432

**Published:** 2024-09-13

**Authors:** Zhenmin Liu, Xingguo Luo, Zhicheng Zhang, Qiang Zhang, Chong Wang, Hongsong Chen, Chunlan Long, Xing Liu, Guanghui Wei

**Affiliations:** a Department of Urology, Children's Hospital of Chongqing Medical University, National Clinical Research Center for Child Health and Disorders, Ministry of Education Key Laboratory of Child Development and Disorders, Chongqing 400014, China; b Chongqing Key Laboratory of Structural Birth Defect and Reconstruction, Chongqing 400014, China

**Keywords:** Apoptosis, CEBPA, Cell cycle, MAFB, Urothelium, Wnt/β-catenin signaling pathway

## Abstract

MAFB is essential for regulating male-type urethral differentiation, and especially, its variation can contribute to hypospadias in mice. However, the potential mechanism is still unclear. Here we observed that the basic leucine zipper (bZIP) transcription factor MAFB and CCAAT/enhancer-binding protein alpha (CEBPA) could promote human urothelium SV-HUC-1 growth. Moreover, MAFB and CEBPA expression were reduced in the prepuce tissues of hypospadias patients. Based on transcriptome sequencing analysis and Western blot, MAFB knockdown was found to suppress CEBPA protein expression and repress Wnt/β-catenin signaling in urothelium cells. Meanwhile, we observed blocked cell-cycle progression from the G1 to the S phase, inhibited cell proliferation, and activated apoptosis. Furthermore, MAFB could facilitate CEBPA transcription and regulate the proliferation of urothelium. The above results indicated that MAFB-mediated inhibition of urothelial SV-HUC-1 growth resulted from inhibiting the Wnt/β-catenin signaling pathway by down-regulating CEBPA. Our findings provide new insight into the understanding of genes associated with hypospadias and the pathogenic mechanism of this disorder.

## Introduction

Hypospadias is manifested by ectopic urethra opening, anomalous penis curvature, and excessive dorsal prepuce, which severely affects the physiological and psychological function of patients.^1^, ^2^, ^3^ Hypospadias occurs in 0.6–69 per 10,000 live births in Asia and increasing reports suggest that the international total prevalence is rising over time.^4^^,^^5^ As one of the most frequent penis congenital malformations, understanding the mechanism of hypospadias has long been a key research focus of reproductive health.^6^^,^^7^ The etiology of hypospadias is commonly considered to be the interaction between genetic and environmental factors.^8^^,^^9^ In this process, genetic variants, especially genes related to the androgen signaling pathway may play a major role.^6^^,^^10^^,^^11^

V-maf musculoaponeurotic fibrosarcoma oncogene homolog B (MAFB), a member of the large bZIP transcription factors, acts as a transcriptional activator of many genes and regulates numerous physiological processes such as cell proliferation, differentiation, and apoptosis.^12^, ^13^, ^14^ In recent years, accumulative studies have reported the association of MAFB with androgen and urethral development.^15^^,^^16^ The MAFB expression in the genital tubercle (GT) of androgen receptor knockout (KO) male mice was much lower than that of wild-type males. MAFB expression was induced in the GT of female mice after androgen treatment. Not only that, MAFB-KO male mice appeared abnormal fusion of the urethra, similar to the hypospadias phenotype. Previous researches indicate that MAFB is crucial for androgen action in male urethra formation.

CEBPA, another bZIP transcription factor, is involved in the proliferation arrest and differentiation of adipocytes and granulocytes,^17^^,^^18^ and its function in the development of the lung and liver during embryogenesis has been studied extensively.^19^, ^20^, ^21^ CEBPA can also be a mediator of epidermal differentiation during development.^22^ So far, whether CEBPA plays a role in urethra formation is not known. Apart from this, the Wnt/β-catenin signaling pathway has already highlighted the importance of development and becomes dysregulated in many diseases.^23^, ^24^, ^25^ It was previously reported that Wnt/β-catenin signaling was inhibited in the dibutyl phthalate-induced hypospadias fetal rat GT.^26^ Additionally, Wnt signaling acts downstream of Shh signaling in the urothelium during GT outgrowth.^27^ Besides, studies have reported that CEBPA could promote the progression of chronic myelocytic leukemia (CML) via the Wnt/β-catenin signaling pathway.^28^ Nevertheless, the role of CEBPA-Wnt/β-catenin axis plays in the urogenital system is not clear.

The endodermal-derived urethral plate epithelium orchestrates the development of GT, which forms the penis or clitoris in later stages.^29^^,^^30^ Both urethra plate epithelium and urothelium are formed from the early fetal urogenital sinus. However, the potential mechanism regarding the impact of MAFB and CEBPA on the urethral epithelia's growth still needs further elucidation. In the current study, we explored the effect of MAFB, CEBPA, and Wnt/β-catenin signaling in the pathogenesis of hypospadias. Our present work could enrich our cognition of the mechanism of the androgen-related gene MAFB in hypospadias formation.

## Materials and methods

### Experimental animals

Mafb-KO mice were constructed through the CRISPR/Cas9 system and facilitated in cooperation with Cyagen Biosciences Inc. (Suzhou, China). Wild-type C57BL/6J mice were purchased from the Laboratory Animal Centre of Chongqing Medical University. The obtained Mafb-KO mice were confirmed by sequencing analysis, and the gene knockout fragment sequence is shown in Figure S1. All mice were maintained in the specific pathogen-free Laboratory Centre of the Children's Hospital of Chongqing Medical University. The homozygous offspring were used for subsequent experiments, and gestational age-matched wild-type mice were used as controls. Ethical approval was given by the Ethics Committee of the Children's Hospital of Chongqing Medical University.

### Human foreskin samples

In accordance with the Ethics Committee of the Children's Hospital affiliated with Chongqing Medical University (Approval Letter No. 318/2022), human foreskin samples were collected with informed consent from patients and their parents. Normal tissue samples obtained from circumcision were taken as controls. Hypospadias tissue samples came from the hypospadias repair. All specimens were kept in liquid nitrogen for the following experiments.

### Scanning electron microscopy

GTs were dissected from GD18.5 (gestational age day 18.5) male Mafb-KO and wild-type mice to visualize morphology. Tissues were fixed in 3.5% glutaraldehyde for at least 48 h and were subjected to gradient dehydration in ethanol dilution (30%, 50%, 70%, 90%, 95%, and 100%). After critical point drying, samples were sputtered with gold and scanned by scanning electron microscopy (FEI Quanta 250, Thermo Fisher, Oregon, USA).

### Hematoxylin-eosin staining

The GD18.5 GT tissues were fixed with 4% paraformaldehyde and embedded in paraffin. Tissues were cut into 4-μm-thickness sections and stained according to the hematoxylin-eosin staining kit (Solarbio, Beijing, China) protocol. The images were taken by the optical microscope (Nikon, Tokyo, Japan).

### Immunohistofluorescence

Immunohistofluorescence staining was performed on paraffin-embedded sections and cells. For paraffin sections, sections were initially deparaffinized and rehydrated and then underwent antigen retrieval. For cell samples, cells were first performed with 4% paraformaldehyde for 20 min and permeabilized with 0.2% Triton X-100 for 10 min. Then, the sections and cells were blocked with 0.5% bovine serum albumin at room temperature for 1 h and incubated with rabbit anti-MAFB antibodies (1:50; Abcam, UK), mouse anti-CEBPA antibodies (1:100; ZenBio, North Carolina, USA), rabbit anti-Wnt5a/b antibodies (1:200; Proteintech, Illinois, USA), and rabbit anti-β-catenin antibodies (1:200; ZenBio) at 4 °C overnight. The following day samples were incubated with FITC- or Cy3-conjugated secondary antibodies (1:200, Proteintech) for 1 h and Hoechst33342 (1:1000, Thermo Fisher) for 30 min away from light. All the immunostained samples were captured with a confocal microscope (Nikon).

### RNA isolation and quantitative real-time PCR (RT-qPCR)

Following collection, the samples were washed once with phosphate buffer saline solution. Total RNA was extracted with the Simply P Total RNA Extraction Kit (BioFlux) and then reverse-transcribed using the RT Master Mix (MCE, New Jersey, USA). Primers used for RT-qPCR are listed in Figure S2. SYBR GREEN qPCR Master Mix (MCE) and CFX96 Real-Time PCR detection system (Bio-Rad, California, USA) were used for reactions. The RT-qPCR parameters were set as follows: 95 °C for 5 min, 40 cycles of 95 °C for 15 s, and 60 °C for 1 min. Results were calculated using the 2^−(ΔΔCt)^ method and based on at least three replicates.

### Cell culture

Human uroepithelial cell line SV-HUC-1 cells were obtained from Haixing Biosciences (Suzhou, China). SV-HUC-1 was cultured in Ham's F–12K medium (Gibco, Thermo Fisher) containing 10% fetal bovine serum, penicillin (100 U/mL), and streptomycin (100 μg/mL). Cells were incubated in the constant temperature incubator at 37 °C and 5% CO_2_.

### Cell transfection

Transfection was performed when the cells reached approximately 50%–60% confluence. Human MAFB-siRNA and CEBPA-siRNA were obtained from Tsingke (Beijing, China). The sequences are listed in Figure S2. Cells were transfected with siRNA and LipofectamineTM3000 (Thermo Fisher) according to the manufacturer's instruction manual, and the final concentration of the siRNA was performed at 50 nM. MAFB-overexpressing lentivirus overexpression vectors, the negative control lentivirus vectors, and polybrene were ordered from HanBio (Shanghai, China). All reagents were prepared and used according to the manufacturer's protocols. In brief, the procedure of cell lines stably overexpressing MAFB went as follows. To infect SV-HUC-1 cells, a culture medium containing MAFB-overexpressing lentivirus (multiplicity of infection = 10), polybrene, and 10% fetal bovine serum was overlaid on the cells for 24 h. The medium was replaced with fresh medium containing puromycin at 5 μg/mL for two days to select stably infected cells (LV-MAFB). As controls, the urothelium cell line SV-HUC-1 cells were infected with a negative control lentivirus (LV-NC), and transfected cells were filtered using puromycin. Total RNA was isolated 48 h after transfection and other experiments were conducted 72 h after transfection. The efficacy of transfection was verified by RT-qPCR and Western blot.

### Western blot

Cellular proteins were extracted with RIPA lysis buffer (MCE), and the concentration was examined via a BCA protein assay kit (Beyotime, Shanghai, China). Multicolor Prestained Protein Ladder WJ103 (Epizyme Biotech, Massachusetts, USA) was used to estimate molecular weight. Proteins were electrophoresed in 10%–15% SDS–PAGE gels (Epizyme Biotech) and transblotted onto PVDF membranes (Millipore, Darmstadt, Germany). After blocked with the protein-free rapid blocking buffer (Epizyme Biotech), the membranes were incubated with mouse anti-β-actin antibody (1:2000; ZSGB-BIO, China), rabbit anti-MAFB antibodies (1:500; Abcam), rabbit anti-CEBPA antibodies (1:1000; Proteintech), rabbit anti-Wnt5a/b antibodies (1:1000; Proteintech), rabbit anti-p-GSK-3β antibodies (1:1000; CST, Massachusetts, USA), rabbit anti-GSK-3β antibodies (1:500; ZenBio), rabbit anti-β-catenin antibodies (1:1000; ZenBio), rabbit anti-cyclin D1 antibodies (1:1000; ABclonal, China), rabbit anti-CDK2 antibodies (1:1000; Proteintech), anti-p21 antibodies (1:1000; Proteintech), rabbit anti-Bcl-2 antibodies (1:1000; ZenBio) and mouse anti-caspase 3/p17/p19 antibodies (1:1000; Proteintech) at 4 °C overnight. After this, blots were incubated with secondary antibodies and developed with Ultra High Sensitivity ECL Kit (MCE) by ChemiDoc Touch Imaging System (Bio-Rad).

### RNA sequencing

Urothelium cell line SV-HUC-1 cells transfected by negative siRNA (si-NC group) and MAFB siRNA (si-MAFB group) (three replicates per group) were sent to Lianchuan Biotech (Hangzhou, China) for RNA sequencing and differential gene expression analysis. Simply put, differentially expressed genes (DEGs) were identified using the R software packages edgeR^31^ and DESeq2.^32^ The selection criteria for DEGs included a log_2_(fold change) of ≥1 or ≤ −1 and a *p*-value <0.05. To perform functional analysis of DEGs, the R software package with the cluster Profiler package was utilized for Gene Ontology (GO) and Kyoto Encyclopedia of Genes and Genomes (KEGG) enrichment analyses, as well as Gene Set Enrichment Analysis (GSEA). In this process, GO and KEGG databases were employed to annotate and analyze DEGs.^33^ To explore functional pathways associated with DEGs, GSEA was employed.^34^ In the revised version, we have added the cells and groups used for RNA sequencing analysis and detailed information about RNA sequencing.

### Cell counting kit-8 (CCK-8) analysis

After transfection, cells were seeded into a 96-well plate of 6000 cells per well and then cultured in an ordinary medium, followed by the addition of 10 μL CCK-8 (MCE) per well at indicated times. Following 2 h of incubation, the optical density value was measured by Synergy LX (Agilent BioTek, California, USA), and the proliferation curve was plotted based on it.

### EdU assay

For EdU cell proliferation staining, EdU Cell Proliferation Kit with Alexa Fluor 488 (BeyoClick) and EdU Cell Proliferation Kit with Alexa Fluor 555 (BeyoClick) were used according to the instructions. Following transfection, cells were treated with 10 μM EdU for 2 h, fixed with 4% paraformaldehyde, and permeabilized in 0.3% Triton X-100 for 15 min. Subsequently, the cells and the Click Reaction Mixture were held for 30 min, and Hoechst 33342 was added for 10 min to visualize the nuclei.

### Flow cytometric analysis

Flow cytometry monitored the cell cycle and apoptosis progression after intervention. Transfected cells in supernatants were collected with adherent cells for apoptosis detection by FITC Annexin V Apoptosis Detection Kit (BD Biosciences, New Jersey, USA) and APC Annexin V (BD Biosciences). For the cell cycle assay, the adherent cells were harvested and fixed with 75% ethanol under 4 °C for at least 24 h. After centrifugation and rehydration, cells were stained with PI/RNase Staining Buffer (BD Biosciences). Samples were analyzed on CytoFLEX (Beckman Coulter, Indiana, USA).

### Statistical analysis

All experiments were independently repeated at least three times, and the data were expressed as mean ± standard deviation. Statistical analysis was performed using GraphPad Prism V.8.0 software. We conducted two-group comparisons using the student's *t*-test. The criteria for statistical significance were set as follows: ∗*p* < 0.05, ∗∗*p* < 0.01, ∗∗∗*p* < 0.005, and ∗∗∗∗*p* < 0.001. The results of the statistical tests were annotated in the figures with asterisks to indicate the level of significance.

## Results

### Changes in hypospadias foreskin and MAFB-KO mouse GT

**Figure 1:**
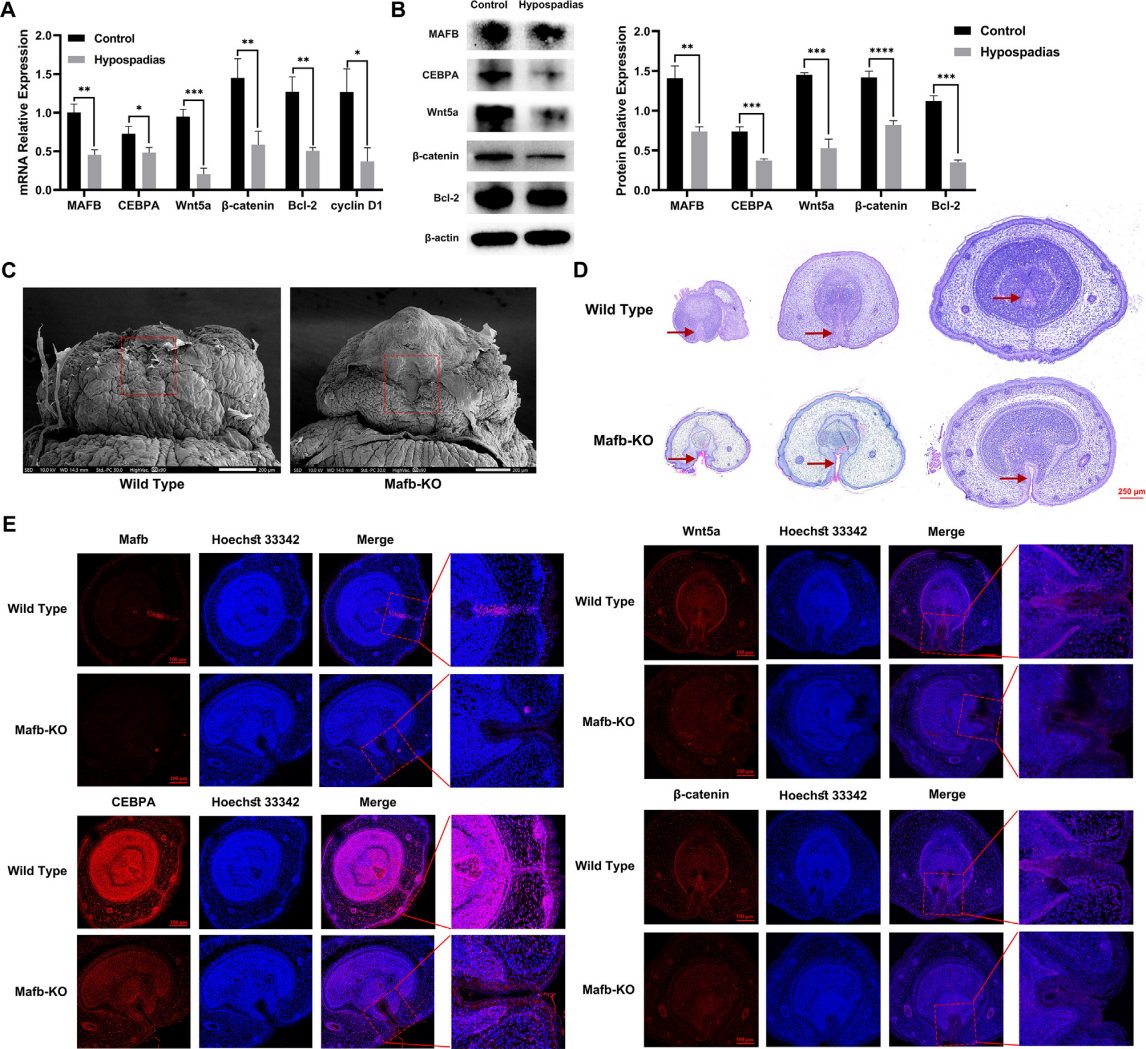
Changes in hypospadias foreskin and Mafb-KO mice GT. **(A)** The mRNA levels of MAFB, CEBPA, Wnt5a, β-catenin, Bcl-2, and cyclin D1 in the foreskin of control and hypospadias groups. **(B)** The protein expression of MAFB, CEBPA, Wnt5a, Bcl-2, and β-catenin in the foreskin of control and hypospadias groups. **(C)** The morphology in the GT of the wild type and Mafb-KO mice. **(D)** The histomorphology of wild-type and Mafb-KO mice. Scale bar = 250 μm. The red arrow represents the urethra. **(E)** The immunofluorescence of Mafb, CEBPA, Wnt5a, and β-catenin in the GT of the wild-type and Mafb-KO mice. Scale bar = 100 μm. All experiments were replicated more than three times. Each bar shows mean ± standard deviation. ∗*p* < 0.05, ∗∗*p* < 0.01, and ∗∗∗*p* < 0.001 versus the control group. KO, knockout; GT, genital tubercle; MAFB, V-maf musculoaponeurotic fibrosarcoma oncogene homolog B; CEBPA, CCAAT/enhancer-binding protein alpha; Wnt5a, Wnt family member 5A; Bcl-2, B-cell lymphoma-2.

We first detected the changes in gene expression of MAFB and associated genes of hypospadias foreskin tissues by RT-qPCR. Compared with normal specimens, the mRNA expression of MAFB, CEBPA, Wnt5a (Wnt family member 5A), β-catenin, Bcl-2 (B-cell lymphoma-2), and cyclin D1 was at significantly low level (Fig. 1A). Based on the above results, we examined the protein expression levels of multiple identified genes, including MAFB, CEBPA, Wnt5a, β-catenin, and Bcl-2, which were in line with data observed above (Fig. 1B).

In addition, we observed the morphology of GD18.5 male GTs in Mafb-KO mice by scanning electron microscopy and hematoxylin-eosin staining. Mafb KO resulted in a significant anomaly of ventral urethral tubulogenesis and severe defects in the prepuce (Fig. 1C, D). We also tested the expression of Mafb, CEBPA, Wnt5a, and β-catenin *in situ* by immunofluorescence staining. A distinct reduction in the expression levels of those proteins was detected in the Mafb-KO GT tissues especially in the urethral area compared with the wild type (Fig. 1E).

The above data indicated that Mafb down-regulation caused hypospadias, and the inhibition of the Wnt/β-catenin signaling pathway was potentially involved in this process.

### The biological function of MAFB in urothelium cell line SV-HUC-1 cells

**Figure 2:**
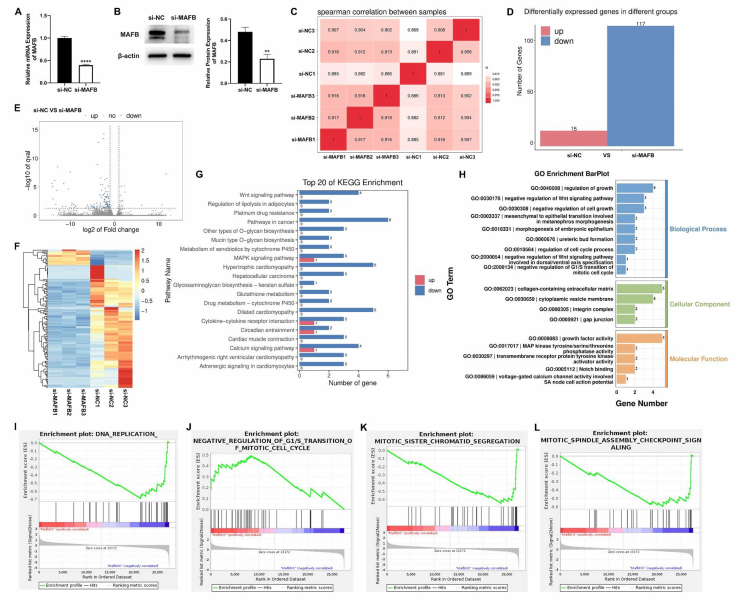
The biological function of MAFB in urothelium cell line SV-HUC-1 cells. **(A, B)** The MAFB mRNA and protein level were measured via quantitative real-time PCR (A) and Western blot (B). **(C)** The Spearman correlation between the control group (si-NC) and MAFB KD group (si-MAFB). **(D)** The DEGs in the control group and MAFB KD group. **(E)** The volcano plot of DEGs in two groups. **(F)** The heatmap of all DEGs in the control group versus the MAFB KD group. **(G)** The top 20 KEGG items of DEGs from the comparison between the control group and the MAFB KD group. **(H)** The DEG GO items in two groups. **(I**–**L)** In the GO enrichment of GSEA analysis, DNA replication (I), negative regulation of G1/S transition of mitotic cell cycle (J), mitotic sister chromatid segregation (K), and mitotic spindle assembly checkpoint signaling (L) were found to be significant. All experiments were replicated more than three times. Each bar shows mean ± standard deviation. ∗∗*p* < 0.01 and ∗∗∗∗*p* < 0.0001 versus control group. MAFB, V-maf musculoaponeurotic fibrosarcoma oncogene homolog B; KD, knockout; DEGs, differentially expressed genes.

To elucidate the role MAFB plays in the uroepithelial cell biological process, we performed RNA sequencing analysis to identify transcriptional changes in MAFB knockdown (KD) urothelium. RT-qPCR and Western blot verified MAFB KD efficiency (Fig. 2A, B). Six samples were clustered by pairwise Spearman correlation in the heatmap (Fig. 2C), and all correlation coefficients were >0.86, which indicated that the two groups of cells have similar sources. In total, 132 DEGs were identified, and the number of down-regulated genes was much higher than the up-regulated (Fig. 2D, E). The heatmap revealed all 132 DEGs (Fig. 2F). To better discover the relevant biological functions, GO, KEGG, and GSEA enrichment analyses were conducted. In the top 20 enriched KEGG items, Wnt signaling pathway was significantly enriched with down-regulated genes (Fig. 2G). Consistent with this observation, the DEGs engaged in the negative control of cell population proliferation, modulation of growth, negative regulation of Wnt signaling pathway, and regulation of the cell cycle process (Fig. 2H). Notably, the GO enrichment of GSEA analysis revealed that DNA synthesis was affected in MAFB KD human uroepithelial cell line SV-HUC-1 cells, such as DNA replication, regulation of G1/S transition of mitotic cell cycle, mitotic sister chromatid segregation, and mitotic spindle assembly checkpoint signaling (Fig. 2I–L).

### MAFB KD induced proliferation inhibition and apoptosis in urothelium cell line SV-HUC-1 cells

**Figure 3:**
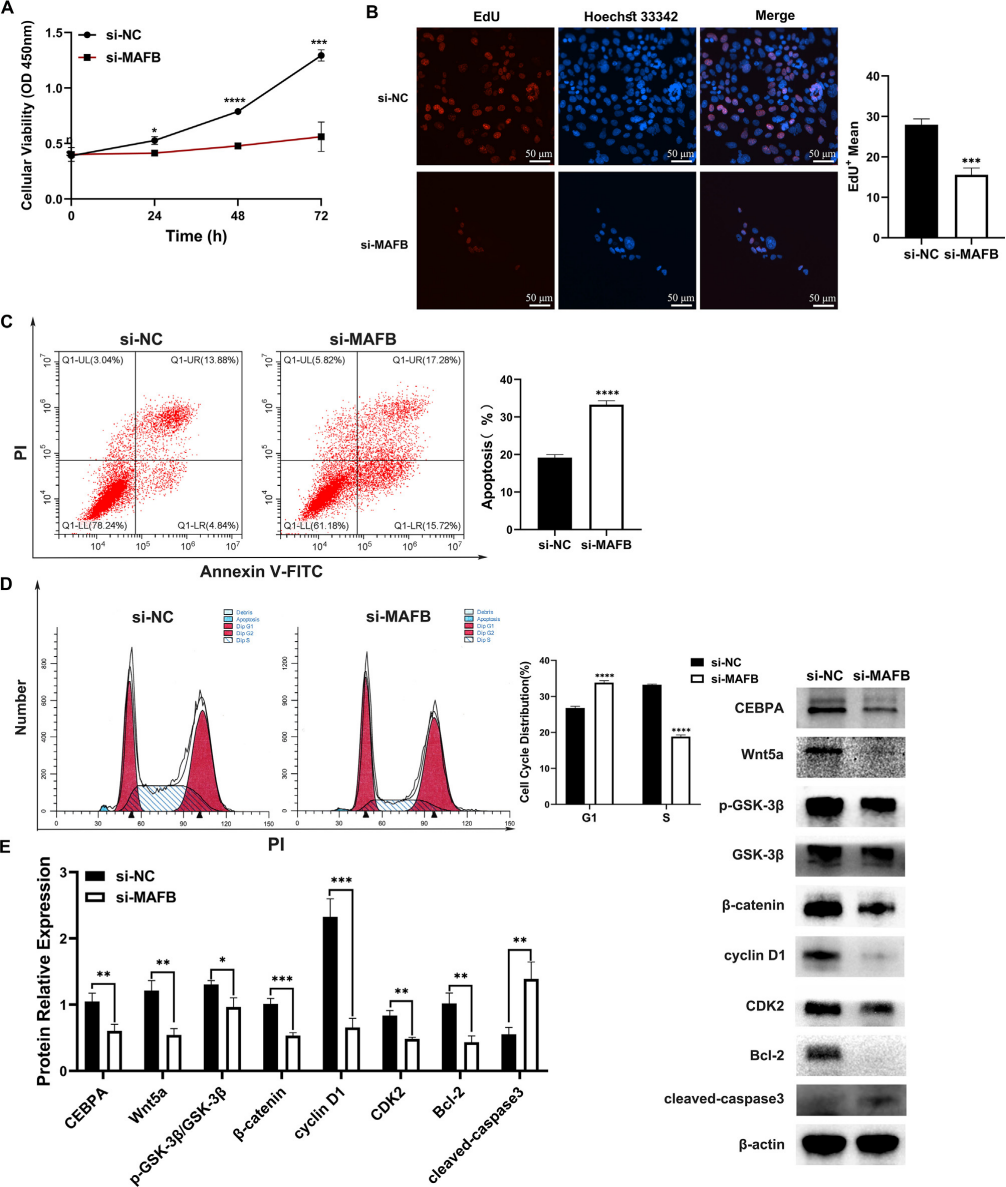
Changes in urothelium cell line SV-HUC-1 cells after MAFB KD. **(A)** The cell proliferation and viability of the control (si-NC) and MAFB KD (si-MAFB) groups were measured by CCK8 assay. **(B)** The cell proliferation levels of the two groups were measured by EdU assay. Scale bar = 50 μm. **(C)** The apoptosis rate of the control and MAFB KD groups. **(D)** The cell cycle changes in the two groups. **(E)** The protein expression of CEBPA, Wnt signaling pathway, cell proliferation, and apoptosis was detected by Western blot. All experiments were replicated more than three times. Each bar shows mean ± standard deviation. ∗*p* < 0.05, ∗∗*p* < 0.01, ∗∗∗*p* < 0.001, and ∗∗∗∗*p* < 0.0001 versus the control group. MAFB, V-maf musculoaponeurotic fibrosarcoma oncogene homolog B; KD, knockout; CEBPA, CCAAT/enhancer-binding protein alpha.

To further examine MAFB function in urothelium cells, we first confirmed that the proliferation of urothelium was inhibited owing to the MAFB KD via CCK-8 and EdU assay (Fig. 3A, B). As shown by flow cytometry, the apoptotic rates of the MAFB KD group (si-MAFB) increased significantly (Fig. 3C). The cell cycle distribution also changed prominently (Fig. 3D). The percentage of cells in the G1 phase increased and the G1-S phase cell cycle was arrested. Together with the previous RT-qPCR and RNA sequencing data, Western blot also showed that the Wnt5a and β-catenin protein expression and p-GSK3β/GSK3β (Glycogen synthase kinase-3 beta) ratio were reduced. The cell-cycle-related protein expression (cyclin D1 and CDK2/cyclin-dependent kinase 2) was observed to be inhibited. Moreover, the protein expression of cleaved-caspase3, apoptosis key factor, was increased and the Bcl-2 expression was inhibited (Fig. 3E). Additionally, the CEBPA level was down-regulated after MAFB KD in SV-HUC-1 cells (Fig. 3E). In all, MAFB KD was observed to inhibit CEBPA protein expression, cell proliferation, and Wnt/β-catenin signaling pathway. Meanwhile, apoptosis and cell cycle arrest were induced by MAFB KD.

### CEBPA KD inhibited cell proliferation, cell cycle, and Wnt/β-catenin signaling pathway in urothelium cell line SV-HUC-1 cells

**Figure 4:**
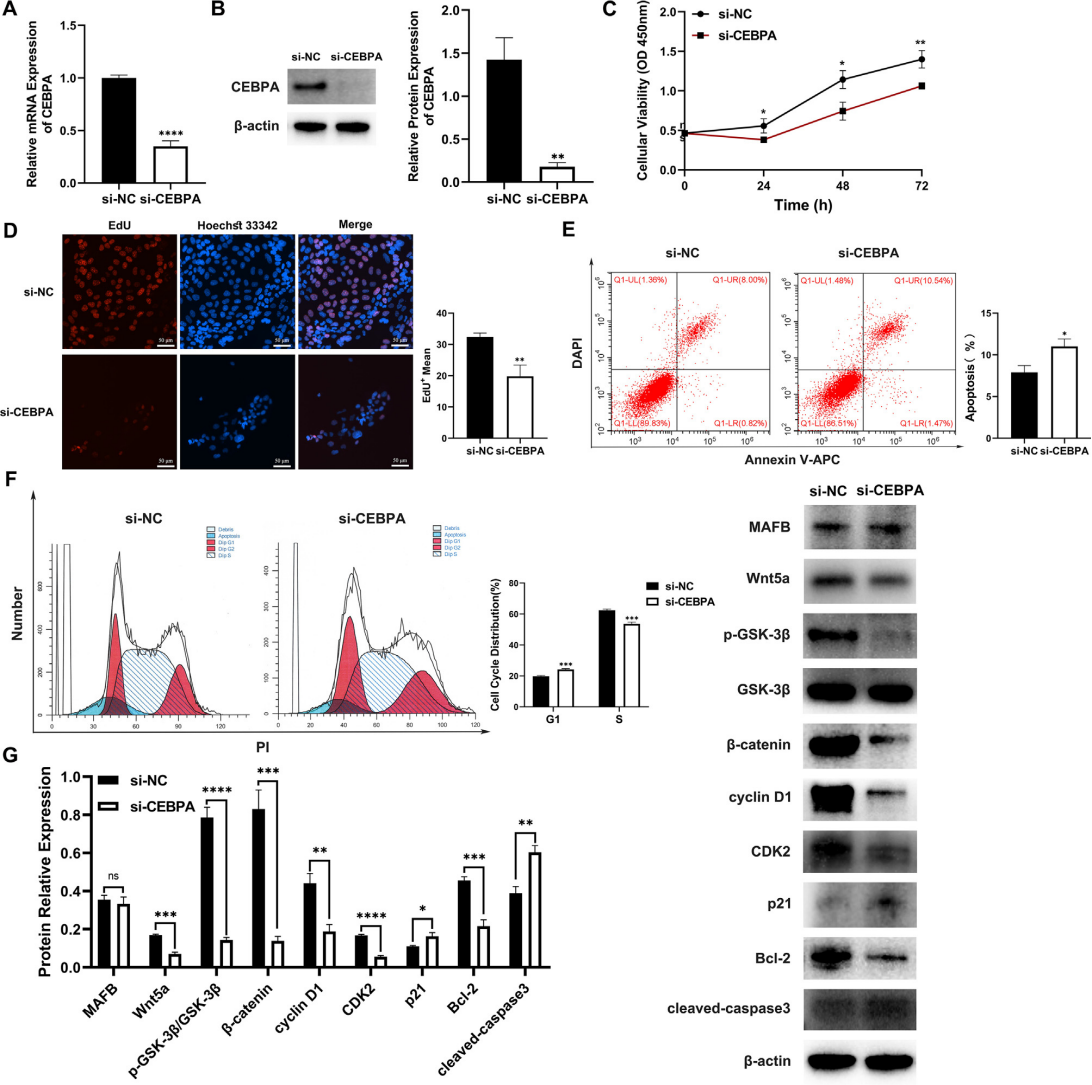
Changes in urothelium cell line SV-HUC-1 cells following CEBPA KD. **(A, B)** The CEBPA mRNA and protein level were measured via quantitative real-time PCR (A) and Western blot (B). **(C)** The cell proliferation and viability of the control (si-NC) and CEBPA KD (si-CEBPA) groups were measured by CCK8 assay. **(D)** EdU assay was employed to detect the cell proliferation levels of the two groups. **(E)** The apoptosis rate of the control and CEBPA KD groups. **(F)** The cell cycle changes of the two groups. **(G)** Western blot was utilized to measure the protein expression of MAFB, Wnt signaling pathway, cell proliferation, and apoptosis. All experiments were replicated more than three times. Each bar shows mean ± standard deviation. ∗*p* < 0.05, ∗∗*p* < 0.01, ∗∗∗*p* < 0.001, and∗∗∗∗*p* < 0.0001 versus the control group. MAFB, V-maf musculoaponeurotic fibrosarcoma oncogene homolog B; CEBPA, CCAAT/enhancer-binding protein alpha; KD, knockout.

To explore the correlation between MAFB and CEBPA and identify the role CEBPA plays in urothelial cells, we employed siRNA transfection to reduce CEBPA protein expression (Fig. 4A, B). Cell proliferation, cell cycle, and the Wnt/β-catenin signaling pathway were inhibited (Fig. 4C–G), whereas MAFB protein expression in the CEBPA KD group (si-CEBPA) was similar to that in the negative control group (si-NC) (Fig. 4G). In summary, CEBPA was indicated to regulate epithelial growth through the Wnt pathway and positively regulated by MAFB.

### MAFB overexpression up-regulated CEBPA protein expression, promoted cell proliferation and Wnt signaling pathway, and inhibited apoptosis in urothelium cell line SV-HUC-1 cells

**Figure 5:**
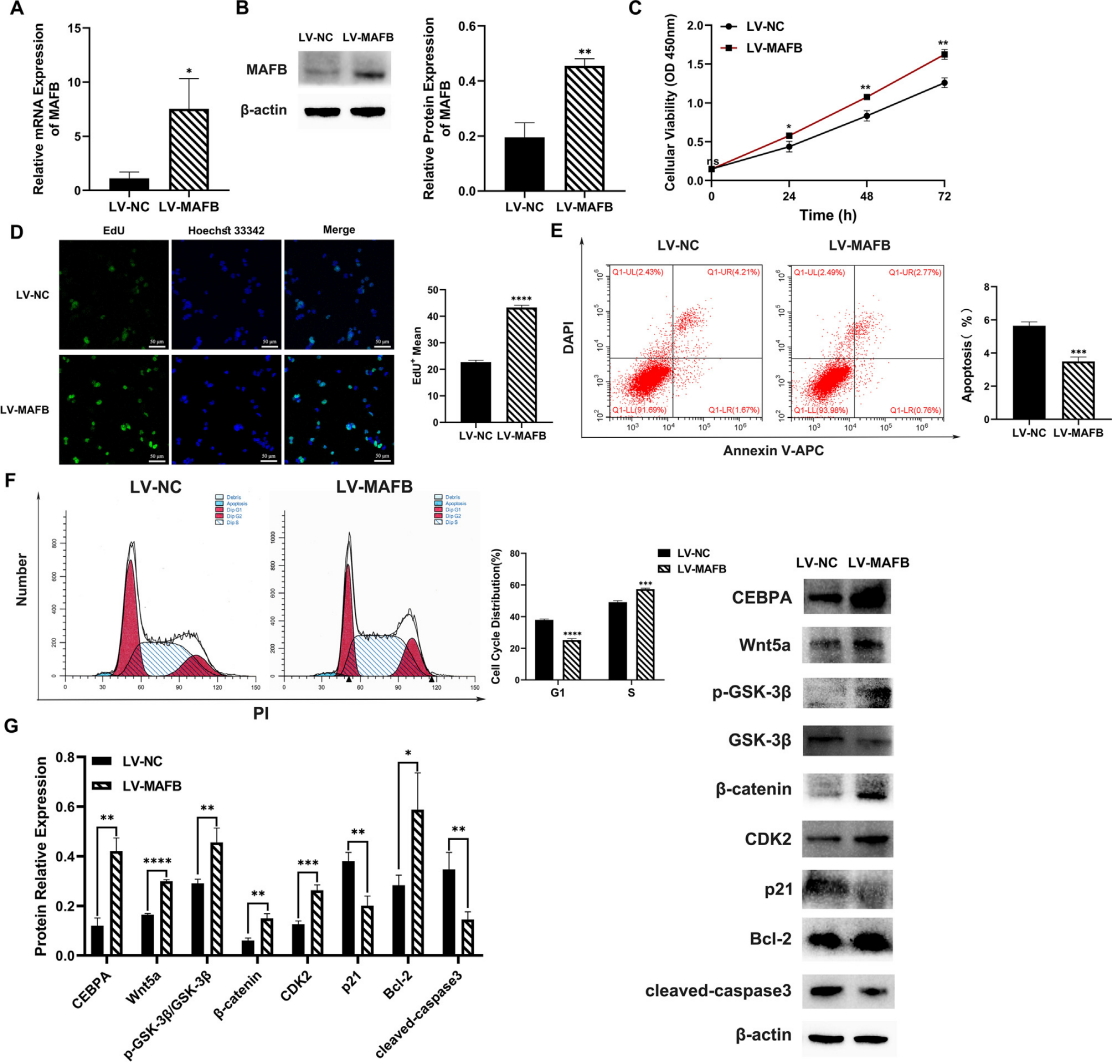
Changes in urothelium cell line SV-HUC-1 cells following MAFB overexpression. **(A, B)** The MAFB mRNA and protein level were measured via quantitative real-time PCR (A) and Western blot (B). **(C)** The cell proliferation and viability of the control (LV-NC) and MAFB overexpression (LV-MAFB) groups were measured by CCK8 assay. **(D)** The EdU assay data of the control and MAFB overexpression groups. **(E)** The cell apoptosis rate in the control and MAFB overexpression groups was measured by flow cytometry. **(F)** The changes in the cell cycle of the two groups. **(G)** The protein expression of CEBPA, Wnt signaling pathway, cell proliferation, and apoptosis was measured via Western blot. All experiments were replicated more than three times. Each bar shows mean ± standard deviation. ∗*p* < 0.05, ∗∗*p* < 0.01, ∗∗∗*p* < 0.001, and ∗∗∗∗*p* < 0.0001 versus the control group. MAFB, V-maf musculoaponeurotic fibrosarcoma oncogene homolog B; CEBPA, CCAAT/enhancer-binding protein alpha.

To further verify the relationship between MAFB and CEBPA, we established stable urothelium cells overexpressing MAFB by lentiviral infection (Fig. 5A, B). Compared with the negative control (LV-NC), cell proliferation in the MAFB overexpression group (LV-MAFB) was significantly increased (Fig. 5C, D). The apoptosis rate in SV-HUC-1 cells was reduced after MAFB overexpression (Fig. 5E). The G1-S phase cell cycle arrest was alleviated by MAFB overexpression (Fig. 5F). The CDK2 expression was increased and p21 was inhibited, indicating activated cell proliferation (Fig. 5G). The Western blot results showed that the protein expression of CEBPA was increased after MAFB overexpression (Fig. 5G). Moreover, the expression of apoptosis key protein cleaved caspase3 was decreased, while the up-regulation of Bcl-2 protein was observed in the MAFB overexpression group (Fig. 5G). The Wnt signaling pathway was also found to be up-regulated in MAFB-overexpressed cells (Fig. 5G). The above data verified that MAFB could increase CEBPA protein expression, promote cell proliferation, activate Wnt signaling pathway, and inhibit apoptosis.

### CEBPA KD reversed the effect of MAFB overexpression and colocalization between MAFB and CEBPA in urothelium cell line SV-HUC-1 cells

**Figure 6:**
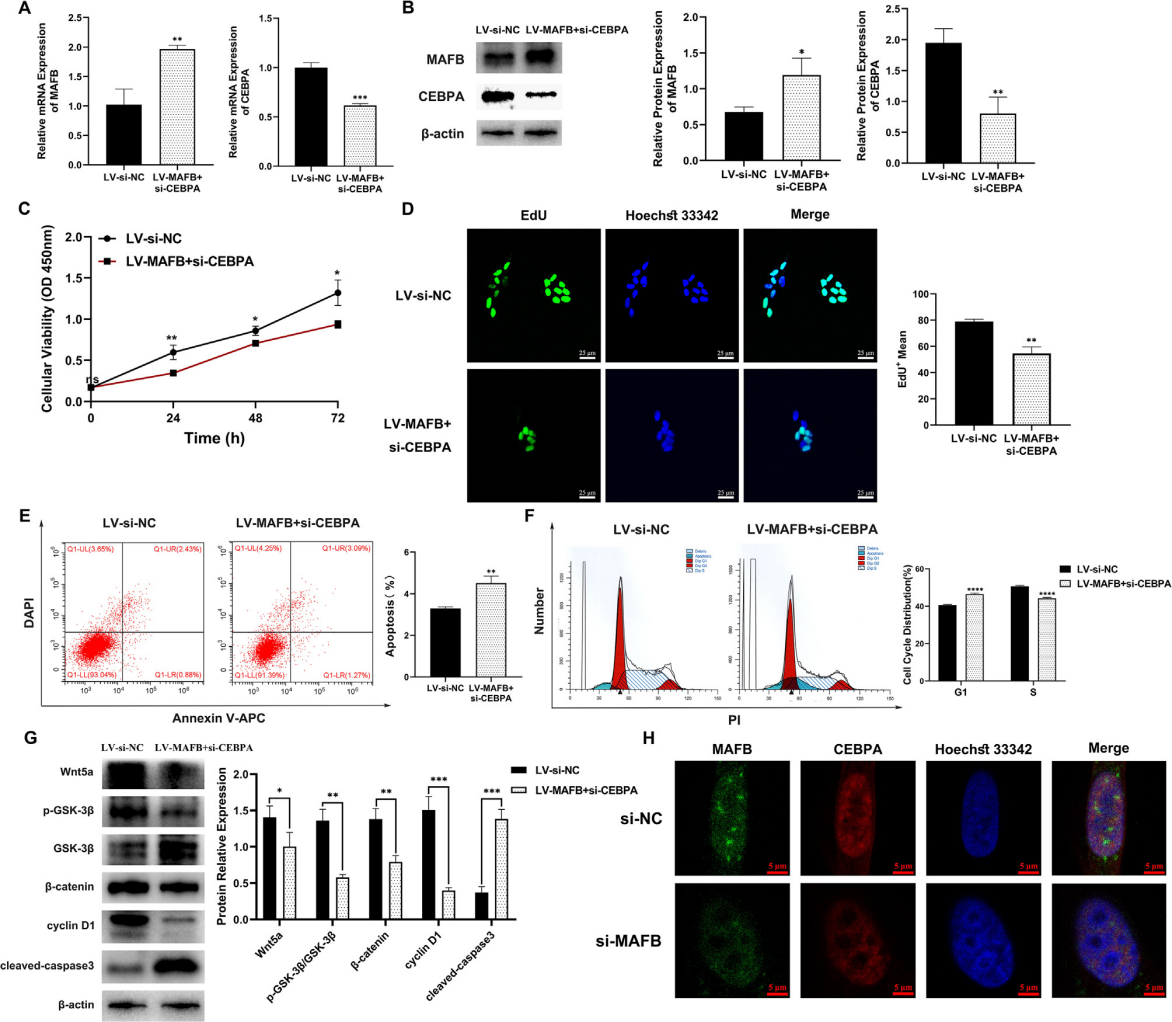
Changes in urothelial cell line SV-HUC-1 with MAFB overexpression + CEBPA KD and MAFB individual KD. **(A, B)** The mRNA and protein level of MAFB and CEBPA were respectively measured by quantitative real-time PCR (A) and Western blot (B). **(C)** The cell proliferation and viability of the control group (LV-si-NC) and MAFB overexpression + CEBPA KD (LV-MAFB + si-CEBPA) group. **(D)** The EdU assay of the control group and the MAFB overexpression + CEBPA KD group. **(E)** The cell apoptosis rate in the control group and the MAFB overexpression + CEBPA KD group was measured by flow cytometry. **(F)** The changes in the cell cycle of the two groups. **(G)** The protein expression of Wnt signaling pathway, cell proliferation, and apoptosis was measured via Western blot. **(H)** The co-location changes of MAFB and CEBPA in the control (si-NC) group and the MAFB KD (si-MAFB) group. All experiments were replicated more than three times. Each bar shows mean ± standard deviation. ∗*p* < 0.05, ∗∗*p* < 0.01, ∗∗∗*p* < 0.001, and ∗∗∗∗*p* < 0.0001 versus the control group. MAFB, V-maf musculoaponeurotic fibrosarcoma oncogene homolog B; CEBPA, CCAAT/enhancer-binding protein alpha; KD, knockout.

To additionally confirm the correlation of MAFB and CEBPA, we also performed a CEBPA siRNA knockdown experiment in MAFB overexpressed SV-HUC-1 cells (LV-MAFB + si-CEBPA group) (Fig. 6A, B). As depicted in Figure 6C–G, compared with the negative control (LV-si-NC group), overexpressing MAFB with CEBPA KD resulted in inhibition of cell proliferation, cell cycle, and Wnt/β-catenin signaling pathway, which suggested that the effect of MAFB overexpression could be reversed by CEBPA knockdown.

Meanwhile, immunohistofluorescence staining showed a strong colocalization of MAFB and CEBPA proteins in SV-HUC-1 cells (si-NC), whereas a weakened signal of CEBPA was observed in the MAFB KD cells (si-MAFB) (Fig. 6H). These results further indicated that MAFB-mediated CEBPA could regulate human urothelium growth through the Wnt/β-catenin signaling pathway.

## Discussion

The occurrence of hypospadias is complex, involving multiple factors. Although the exact pathogeny is still unclear, previous studies have revealed both environmental and genetic abnormalities are deeply involved.^6^^,^^35^, ^36^, ^37^ Environmental endocrine disruptors can contribute to hypospadias by affecting hormonal signaling pathways that are involved in sexual differentiation and may be associated with gene–environment interactions. Additionally, about 30% of cases have been proven to present specific genetic mutations.^38^ Ultimately, all these factors could influence the expression of key genes involved in urethra formation. Further studies are needed to deepen the knowledge about this process.

During normal penile development, the urethral plate epithelium, which extends distally to the tip of the glans, is found in the GD10.5 embryonic penis. Until GD18.5, the lumen formed by the urethral plate opens at the glans to form a normal urethral meatus.^39^ Both human and mouse prepuce development results from the initial epithelial–epithelial contact and ventral fusion of the prepuce folds.^40^ In this process, hypospadias may occur if any factors disturb epithelial fusion. Besides, urothelial tissue reconstruction is also the focus of tissue engineering technology in the repair of hypospadias.^41^, ^42^, ^43^ As uroepithelium is of great significance in the urethra morphogenetic process, more tailored studies focused on the molecular mechanism are needed. In this research, urothelium cell line SV-HUC-1 cell was selected as the major subjects in the study, and two important factors in the urethral epithelium, MAFB and CEBPA, were focused. We demonstrated the changes in the expression of MAFB, a key mediator of androgen action in the process of urethral masculinization, and other related genes in the prepuce of hypospadias patients. Furthermore, we discovered the correlation between MAFB and CEBPA in urothelial cells and their effects on urothelium growth via the Wnt/β-catenin signaling pathway. As a special multi-layer epithelium, urothelial not only proliferates and differentiates during development but also triggers strong regenerative capacity after postnatal injury.^44^^,^^45^ We believe that an in-depth understanding of the key regulatory factors and pathways in urethra epithelial proliferation and growth would contribute to the repair of hypospadias.

The role MAFB plays in hypospadias and the potential mechanism is intriguing. The dynamic changes of MAFB have been previously observed in urethral formation.^46^ However, these studies were performed in mesenchyme rather than tested MAFB KO in epithelium. In previous studies, MAFB-KO male mice manifested significant urinary tract defects in the late embryonic stage.^15^ In addition, apoptosis was activated and the proliferation was inhibited in the hindbrain choroid plexus epithelial cells of MAFB deficient mice.^47^ In the pancreas, MAFB gene deletion impeded both G1/S and G2/M cyclin expression and blocked β-cell expansion.^48^ MAFB was also reported to promote the proliferation of ovarian cancer cells.^49^ In our research, consistent with the gene changes observed in RNA sequencing, including DNA replication and G1/S transition, the arrest of the G1/S transition cycle in human urothelium was observed in flow analysis after MAFB KD. Consequently, MAFB was found to promote proliferation and cell cycle in urothelium cells. As a bZIP protein, Mafb dimerizes with other bZIP proteins to bind to specific DNA sequences, thereby regulating the transcription of target genes involved in various cellular processes, including differentiation, proliferation, and apoptosis,^50^^,^^51^ which is relevant to our other findings. These results highlighted that MAFB was pivotal in the urothelial development and hypospadias occurrence.

As a transcription promoter/enhancer with a bZIP domain, CEBPA is significant in general growth and development and tumorigenesis. To our knowledge, it is the first time CEBPA has been reported to be correlated with genital development and hypospadias, as the CEBPA mRNA expression was found to be down-regulated in human foreskin tissue. Additionally, CEBPA regulates cell proliferation by modulating cell cycle machinery and controlling apoptosis.^52^, ^53^, ^54^ In hepatocellular carcinoma, CEBPA KD suppressed cell growth which is indicated by decreased cyclin A and CDK4 expression.^52^ CEBPA is also crucial for respiratory epithelial differentiation and regeneration.^55^^,^^56^ In our results, CEBPA KD disturbed urothelial growth, induced G1/S phase transition arrest, and activated apoptosis. Moreover, we also demonstrated a robust association may exist between MAFB and CEBPA in the urothelium. These results expand our understanding of hypospadias pathogenesis. Apart from that, since CEBPA could promote epithelial–mesenchymal transition through the nuclear translocation of β-catenin in hepatocellular carcinoma,^19^ and given the vital role of epithelial–mesenchymal transition in urethral plate fusion during normal urethral development,^57^ we plan to probe the effect of CEBPA down-regulation on the urethral epithelial–mesenchymal transition at a later stage. Besides, the potential interactions between CEBPA and other signaling pathways in genital development shall be investigated to provide a more comprehensive understanding of its functions. Meanwhile, CEBPA is important in controlling T cell immunodepression in cell senescence, which suppressed cyclin D1, demonstrated a strong antiproliferative effect, and promoted the expression of proinflammatory cytokine.^58^^,^^59^ It has also sparked our interest in cellular senescence and inflammation in urothelium, which could provide deeper insights into the pathogenesis of hypospadias.

It has been reported previously that the Wnt/β-catenin signaling pathway has a notable role in embryo development, especially in the development of embryonic external genitalia.^27^^,^^60^, ^61^, ^62^ Wnt/β-catenin pathway is essential to maintain endodermal urethral homeostasis, and hypospadias occurred in both endodermal and ectodermal β-catenin-KO mice.^62^ Several Wnt7a-related genes are regulated by the androgen pathway, which can be involved in the androgen-dependent development of GT.^63^ CEBPA also promoted the development of chronic myelocytic leukemia by glycogen synthase kinase-3 (GSK3) and Wnt/β-catenin signaling.^28^ Additionally, it can regulate ovarian cancer cell proliferation through modulation of Wnt signaling.^64^ Our clinical data illustrated that the expression level of representative genes of the Wnt/β-catenin pathway was decreased in human prepuce. Furthermore, *in vitro* experiments demonstrated that MAFB and CEBPA regulated uroepithelium growth by activating Wnt/β-catenin signaling. These results further highlighted Wnt/β-catenin signaling pathway was critical in hypospadias occurrence.

Cell proliferation, apoptosis, and migration play a multiple fundamental role in mammalian development, and impairment of the apoptosis-proliferation balance can contribute to structural abnormalities. In normal human fetal penile development, cellular proliferation markers were clearly elevated on the ventral side relative to the dorsal part, where the urethral plate forms and canalizes, while apoptotic markers were rarely noted in the developing penis.^65^ Several factors, such as environmental and genetic background, could disrupt this balance in various types of cells in urethra development.^57^^,^^66^, ^67^, ^68^ Isl1 (ISL LIM homeobox 1) deletion inhibited urethral epithelium differentiation and caused significantly increased cell death in the urethra.^66^ Silencing Rab25 would impede foreskin fibroblast cell proliferation and migration.^69^ In fact, the same factor may have different roles within different types of cells and even within different cells of the same type. Mafb promoted apoptosis by inhibiting arteriosclerosis of foam cells through mediating the oxidized low-density lipoprotein-activated liver X receptors/retinoid X receptors,^70^ and MAFB promoted cancer stemness growth in osteosarcoma through up-regulation of Sox9.^12^ In this work, the specific role of MAFB in urethral epithelium was investigated, which advances our understanding of the contribution of heritable factors to hypospadias.

Nevertheless, some intriguing questions need to be answered in our future research. First, although MAFB was found to be an upstream regulator of CEBPA in SV-HUC-1 cells, the precise molecular mechanism in this regulation is unclear. Whether MAFB immediately interacts with CEBPA or whether additional factors are involved in this regulation will be investigated in detail in our future work, and they include but are not limited to CO-IP or luciferase experiments. Second, due to the lack of urethral plate epithelium, human urothelial SV-HUC-1 cells were employed in this study as a reasonable substitute. In the future, we will combine spatial transcriptome and single-cell sequencing to get more representative cell lines to address this question. Specific marker genes will be identified to screen the urethral plate epithelium and provide new insights for the following exploration. Third, whether CEBPA KO results in damaging urethral integration directly is still unknown. CEBPA-KO mice will be utilized in our follow-up studies.

In conclusion, our present work reported CEBPA as a novel gene involved in the pathogenesis of hypospadias. Moreover, abnormal expression of MAFB and CEBPA affected uroepithelial growth by Wnt/β-catenin signaling. These findings may offer a deeper understanding of normal penile development and the occurrence of hypospadias.

## Funding

This work was financed by the 10.13039/100014717National Natural Science Foundation of China (No. 81970571), the Natural Science Foundation of Chongqing Municipality, China (No. CSTB2022NSCQ-MSX1001), and the Program for Youth Innovation in Future Medicine, Chongqing Medical University (No. W0109).

## Data availability

The data are available from the corresponding author upon reasonable request.

## CRediT authorship contribution statement

**Zhenmin Liu:** Conceptualization, Methodology, Writing – original draft. **Xingguo Luo:** Data curation, Software. **Zhicheng Zhang:** Conceptualization, Data curation, Software. **Qiang Zhang:** Data curation, Software. **Chong Wang:** Investigation, Visualization. **Hongsong Chen:** Investigation, Visualization. **Chunlan Long:** Writing – review & editing. **Xing Liu:** Writing – review & editing. **Guanghui Wei:** Writing – review & editing.

## Conflict of interests

The authors declare that they have no known competing financial interests or personal relationships that could have appeared to influence the work reported in this paper.
