# Supplementary material for: MAFB-mediated CEBPA regulated human urothelium growth through Wnt/β-catenin signaling pathway
Source: Genes Dis. 2024 Sep 13;12(1):101432. doi: 10.1016/j.gendis.2024.101432 (PMC11577151; doi:10.1016/j.gendis.2024.101432)
Supplement: Multimedia component 1 [file mmc1.docx]

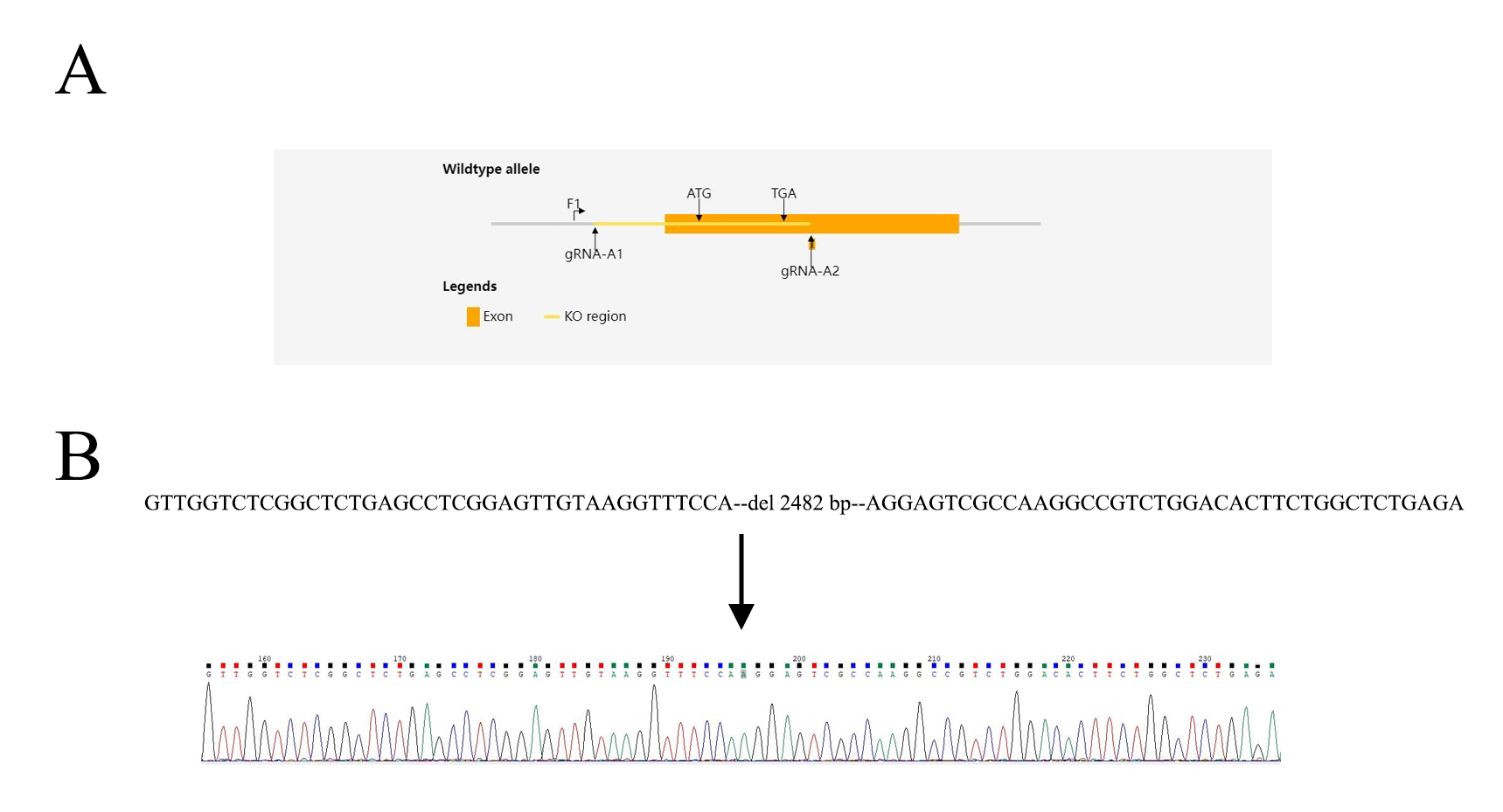


**Figure S1 The DNA sequence validation of Mafb KO mice.**

A. The DNA product obtained from PCR amplification was sent to the first-generation sequence to confirm the targeting situation. F1: 5’-CAAAGAACAAACCGGTTCAGGG-3’. gRNA-A1: CCTTTCACTTGCCAGTCGTGTGG. gRNA-A2 : ACTCCTTGGAATAAGCCTCGCGG. B. The mouse sequencing result showed deletion of 2482 bp.
