# Supplementary material for: MAFB-mediated CEBPA regulated human urothelium growth through Wnt/β-catenin signaling pathway
Source: Genes Dis. 2024 Sep 13;12(1):101432. doi: 10.1016/j.gendis.2024.101432 (PMC11577151; doi:10.1016/j.gendis.2024.101432)
Supplement: Multimedia component 2 [file mmc2.docx]

**Supplementary material 2. Primers used in this study.**

| Genes | primers | Sequences (5’-3’) |
| --- | --- | --- |
| Mafb | P01 | GACGCAGCTCATTCAGCAG |
|  | P02 | CTCGCACTTGACCTTGTAGGC |
| CEBPA | P03 | GCCATGGCACCGGATACCA |
|  | P04 | TGGCAAGTATCCGAGCAAAA |
| Wnt5a | P05 | ATTCTTGGTGGTCGCTAGGTA |
|  | P06 | CGCCTTCTCCGATGTACTGC |
| β-catenin | P07 | GCCAAGTGGGTGGTATAGAG |
|  | P08 | GGGATGGTGGGTGTAAGAG |
| Bcl-2 | P09 | GGTGGGGTCATGTGTGTGG |
|  | P10 | CGGTTCAGGTACTCAGTCATCC |
| Cyclin D1 | P11 | TGATACCAGAAGGGAAAGC |
|  | P12 | GCTCAGGGTTATGCAAATAC |
| β-actin | P17 | TAGTTGCGTTACACCCTTTCTTG |
|  | P18 | CACCTTCACCGTTCCAGTTTT |
| Mafb-siRNA | P19 | GCAACUACCAGCAGAUGAATT |
|  | P20 | UUCAUCUGCUGGUAGUUGCTT |
| CEBPA-siRNA | P21 | CAGAGAGCUCCUUGGUCAA(dT)(dT) |
|  | P22 | UUGACCAAGGAGCUCUCUG(dT)(dT) |
